# Long non-coding RNA involved in the carcinogenesis of human female cancer - a comprehensive review

**DOI:** 10.1007/s12672-025-01848-1

**Published:** 2025-02-06

**Authors:** Nazia Afroze, Madhumitha K. Sundaram, Shafiul Haque, Arif Hussain

**Affiliations:** 1 School of Life Sciences, Manipal Academy of Higher Education, Dubai Campus, P.O. Box 345050, Dubai, United Arab Emirates; 2 Department of Nursing, College of Nursing and Health Sciences, Jazan University, Jazan, Saudi Arabia; 3 School of Medicine, Universidad Espiritu Santo, Samborondon, Ecuador

**Keywords:** Cancer, lncRNA, siRNA, RNA-based, Phytochemicals, Therapeutics, Diagnostics, Gynaecological

## Abstract

Recent years have seen an increase in our understanding of lncRNA and their role in various disease states. lncRNA molecules have been shown to contribute to carcinogenesis and influence the various cancer hallmarks and signalling pathways. It is pertinent to understand the specific contributions and mechanisms of action of these molecules in various cancers. This review provides an overview of the various lncRNA entities that influence and regulate the gynaecological cancers, namely, cervical, breast, ovarian and uterine cancers. The review curates a list of the key players and their effect on cellular processes. lncRNA molecules show immense potential to be used as diagnostic and prognostic indicators and in therapeutic strategies. Several phytochemicals, small molecules, RNA-based regulators, oligos and gene editing tools show promise as a therapeutic strategy. While this review highlights the promising developments in this field, it also underscores the necessity for further research to delineate the complex role of lncRNAs in cancer.

## Introduction

Non-coding RNAs (ncRNAs), despite not encoding proteins, have a significant role in gene regulation and cellular function and influence both normal physiology and cancer. There are several kinds of ncRNAs. MicroRNAs (miRNAs) are single-stranded RNAs about 21–25 nucleotide in length derived through the Drosha-Dicer pathway. They regulate gene expression by binding to the 3′ untranslated regions of target mRNAs, resulting in translational repression. They influence development and differentiation, and by acting as tumor suppressors or oncogenes is linked with carcinogenesis (Peng et al.). Circular RNAs (circRNAs) are closed circular entities shaped by back-splicing of pre-mRNAs. They can act as miRNA sponges, regulate transcription, and may also encode small peptides. Their aberrant expression can influence cancer pathways (Conn et al., li Jiao et al.). Small Nucleolar RNAs (snoRNAs), sized at about 60–300 nucleotides, regulate several post-transcriptional processes, and influence chemical modification of rRNA and snRNA. Dysregulation can impact systemic changes leading to altered cellular metabolism and cancer progression (Bratkovic et al.). Piwi-Interacting RNAs (piRNAs) are 24–31 nucleotides long RNA molecules with 3′ end, 2′-O-methyl modification. These PIWI protein binding RNA molecules can regulate proceses at the transcriptome and proteome level. Their aberrant expression has been linked to cancer onset and hallmarks (Chen et al.). Small Interfering RNAs (siRNAs) are double-stranded RNAs of about 20–23 nucleotide length produced by Dicer action. siRNA silences specific mRNAs and influences cellular processes. Their utilization in therapeutic approaches has yielded significant results (Charbe et al.). Transfer RNA-derived small RNAs (tsRNAs) derived from tRNA cleavage and ribosomal RNA-derived small RNAs (rsRNAs), derived from rRNA cleavage are recent members of the ncRNA family. They influence gene regulation, stress responses, and signalling pathways and are associated with cancer physiology (Li et al.). Long non-coding RNAs (lncRNAs) are a category of RNA molecules that are greater than 200 nucleotides in length and do not encode for proteins. The total number of lncRNAs transcripts is projected to be between 30,000 and 60,000 in number [1]. They have been shown to play important regulatory roles in gene expression, chromatin remodelling, post-transcriptional regulation and influence various cellular processes [2].

lncRNAs impact regulatory networks at various molecular levels and regulate gene expression at chromatin, transcriptional, and post-transcriptional levels, influencing critical pathways in cancer progression and influence multiple cellular processes simultaneously. The specific expression of lncRNAs in cancer tissues makes them ideal therapeutic targets and can inform strategies to overcome resistance. lncRNAs are stable in biofluids and show specific expression patterns in cancer that facilitate early detection and disease monitoring. lncRNAs represent an area of research that can lead to milestone developments in carcinogenesis and therapeutics and is therefore the focus of this study.

### lncRNA, its biogenesis, regulation and mode of action

The biogenesis of lncRNAs is like that of protein-coding mRNAs, involving transcription, processing, and maturation. lncRNAs are transcribed by RNA polymerase II, producing primary transcripts or pre-lncRNAs which undergo post-transcriptional modifications, such as 5′ capping, splicing, and 3′ polyadenylation. The resulting mature lncRNAs are then transported to different cellular compartments, where they perform their regulatory functions. LncRNAs are classified as exonic, intronic, overlapping, intergenic or anti-sense based on their start point and location [3]. Long non-coding RNAs can be transcribed from promoters of protein-coding genes, enhancer regions, intergenic regions and bidirectional promoters [4]. The regulation of lncRNAs is complex and involves a variety of mechanisms at different stages of their biogenesis and function. The transcription of lncRNAs can be regulated by transcription factors and epigenetic modifications, such as DNA methylation and histone modifications. The splicing of lncRNAs can be regulated by splicing factors, which can modulate the inclusion or exclusion of specific exons. The stability and degradation of lncRNAs can be regulated by RNA-binding proteins and microRNAs. The localization of lncRNAs to specific subcellular compartments can also be regulated by RNA-binding proteins. The function of lncRNAs can be regulated by their interaction with other RNA molecules, proteins, and DNA [5].

Long non-coding RNAs regulate gene expression by cis- and trans-regulatory processes. Cis acting lncRNAs influence the expression of genes that are alongside their own locations on the genome; whereas trans-acting moieties can move to other locations within the cell and exert their influence [5, 6]. These RNAs can exert their influence at all points of the gene expression spectrum, acting on the transcriptional, translational, and post-translational levels and interact with DNA, RNA as well as proteins [7, 8]. lncRNAs can alter the chromatin structure and influence histone modification [9]. Their ability to adopt various secondary structures allows them to function as scaffolds and enhancers [6]. They are also able to bind to transcription factors as decoys or recruit transcription factors to target areas [10]. They have also been shown to interact with a wide array of transcription related proteins. They can influence the activity of miRNA, stop their transcription and influence their stability [11]. They can also inhibit the interaction of miRNAs with the target mRNAs by functioning as competitive endogenous RNAs [12]. Through these various actionable areas, they can influence several biological processes such as proliferation, development, differentiation, DNA repair, and cell fate [5]. Dysregulation of their expression and action is associated with several health and disease states including carcinogenesis.

### Overview of its role in carcinogenesis of common cancers

**Fig. 1:**
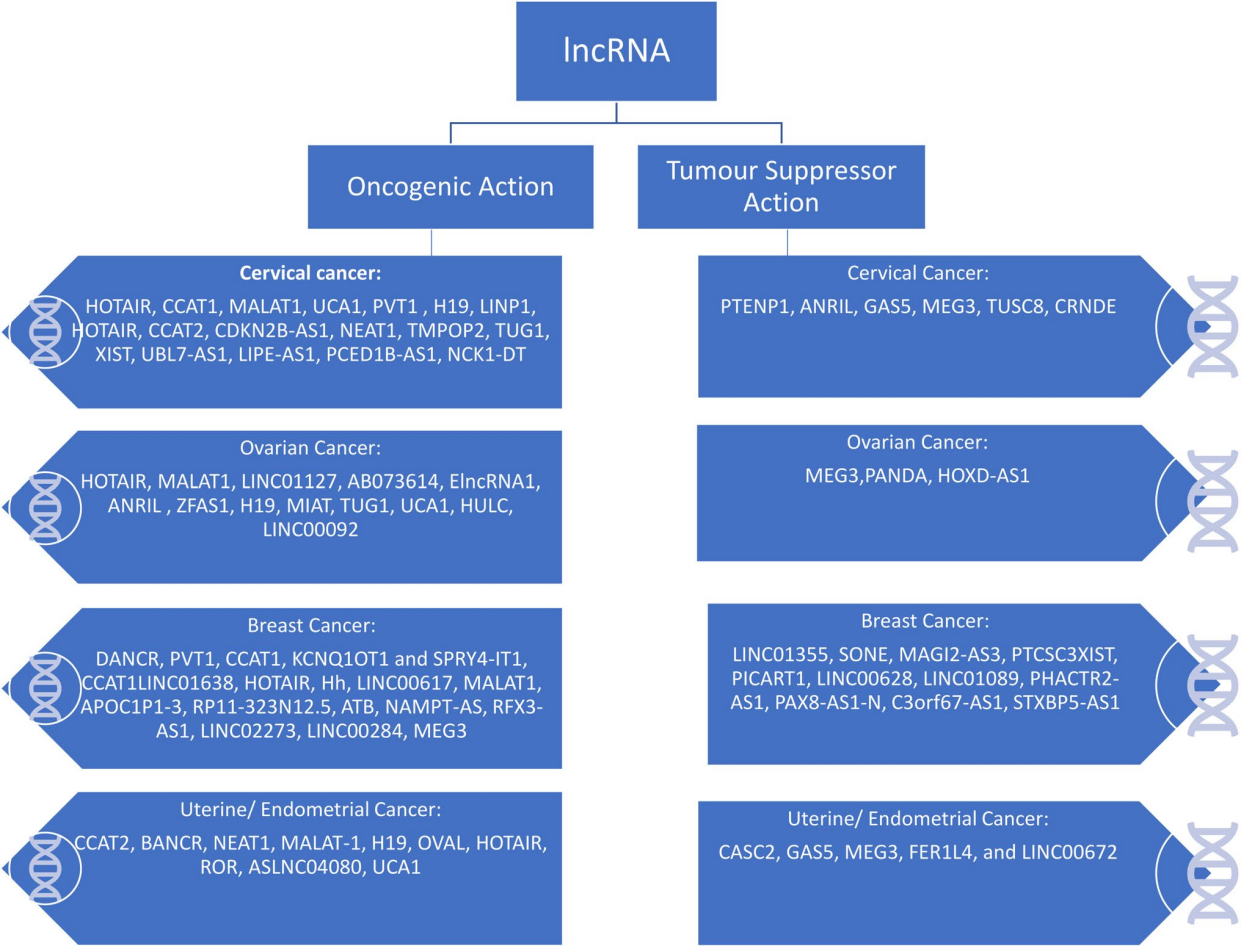
Types of lncRNA in cancer. The image highlights some of the common and well-studied lncRNAs displaying both oncogenic and tumour suppressor function in gynecological cancers

Recent research has highlighted the role of lncRNAs in the development and progression of cancer. lncRNAs have been shown to contribute to various hallmarks of cancer including proliferation, evasion of metastasis, modulation of metabolic pathways, invasion and metastasis, immune-modulation and lead to tumour progression. In several cancers the major reason has been attributed to the differential expression and dysregulation of lncRNA [5]. The upregulation of lncRNA with oncogenic potential such as HOTAIR, MALAT1, PCAT1, H19 have been reported in various cancer types and have been associated with various processes contributing to cancer development. Several lncRNAs have been shown to play a role in breast cancer development and progression [13–16]. HOTAIR is a lncRNA that is upregulated in breast cancer and promotes tumour growth and metastasis by repressing the expression of tumour suppressor genes [17]. MALAT1, is associated with poor prognosis in breast cancer patients and promotes tumour cell migration and invasion [13]. In prostate cancer, PCAT-1, a lncRNA is upregulated and promotes tumour growth and metastasis by regulating the expression of genes involved in cell cycle progression and apoptosis [18]. PRNCR1, is associated with aggressive prostate cancer and regulates the expression of genes involved in androgen receptor signalling [19]. Several lncRNAs including MALAT1, have been shown to play a role in the development and progression of lung cancer and is associated with poor prognosis in lung cancer patients [14]. Upregulated H19 promotes cell proliferation and invasion, while HOTAIR, promotes tumour growth, metastasis and angiogenesis and is associated with poor prognosis in colorectal cancer [16] (Fig. 1).

**Fig. 2:**
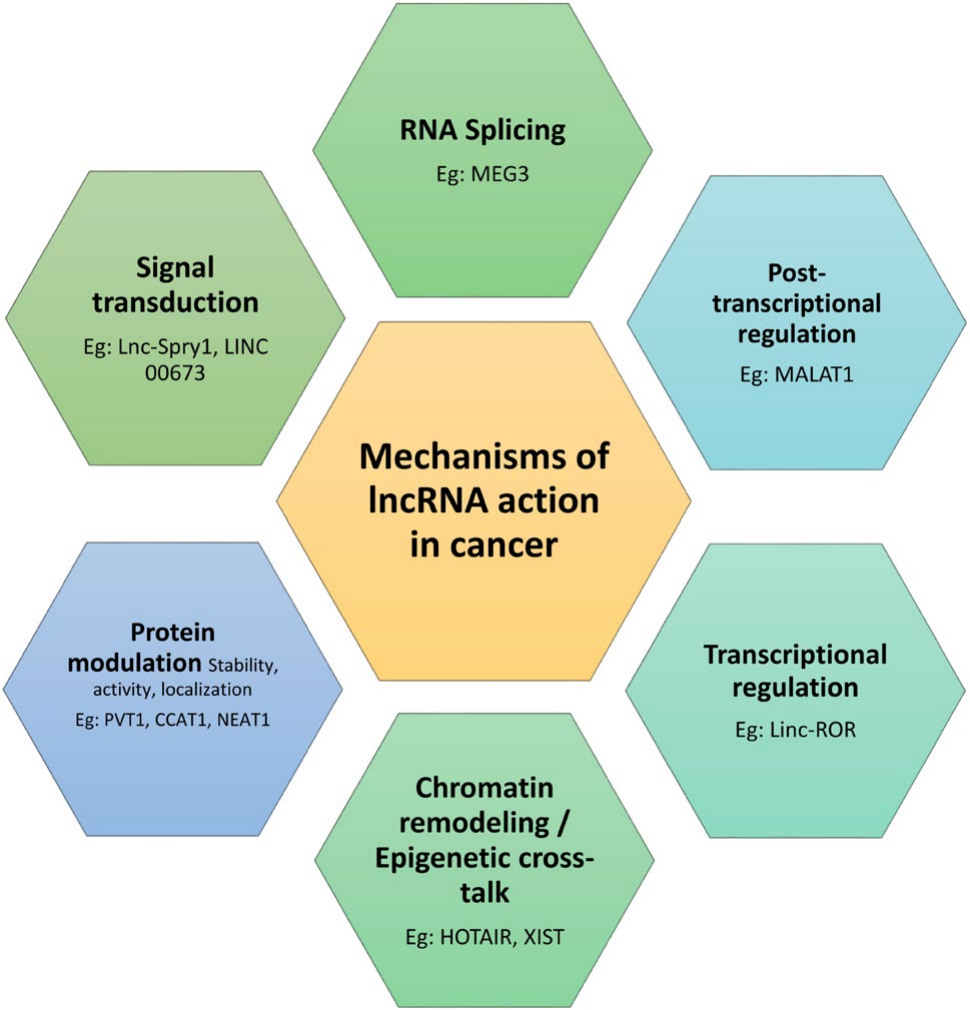
Mechanisms of lncRNA action in cancer. The general mechanisms of actions adopted by lncRNAs in mediating carcinogenic processes are shown

The downregulation of several lncRNA with tumour suppressor activity such as BGL3, GAS5, MEG3, NBAT-1 and PTENP1 have been reported in different cancers [20–24]. TINCR was reported to have a low expression in prostate cancer, and was found to be associated with advanced disease, lymph spread, metastasis and poor prognosis [25]. TSLNC8 has been reported to be downregulated in lung cancer cells, while its increased expression promotes apoptosis and lowers migration and proliferation [26]. GAS5 is a very important lncRNA that functions as a tumour suppressor in several cancers including lung, breast, gastric, cervical, ovarian, renal, kidney, oesophagus and colorectal (reviewed in [27]). It has been shown to influence a wide range of cellular activities and predict patient outcome and is therefore, a prominent diagnostic and prognostic marker. A list of the most important and well-studied lncRNA in gynaecological cancers having oncogenic and tumour suppressor activity is shown in Fig. 1 and the common mechanisms of action in carcinogenesis is shown in Fig. 2.

Long non-coding RNAs have emerged as promising biomarkers and diagnostic indicators in cancer. lncRNAs are easily detectable and stable in body fluids (plasma, urine, saliva) and tissue biopsies and can be measured using minimally invasive techniques [28, 29]. Several population-based studies have been able to categorize signatures of lncRNA that reflect disease severity, FIGO classification, metastatic potential, tumour growth, recurrence risk, as well as survival and prognostic outcomes [30–32]. The US FDA has approved a urine-based detection of PCA3 in prostate cancer [33]. Its high expression is observed in aggressive cancers and hence it also serves as a prognostic indicator [34].

### Therapeutic approaches based on lncRNA

**Fig. 3:**
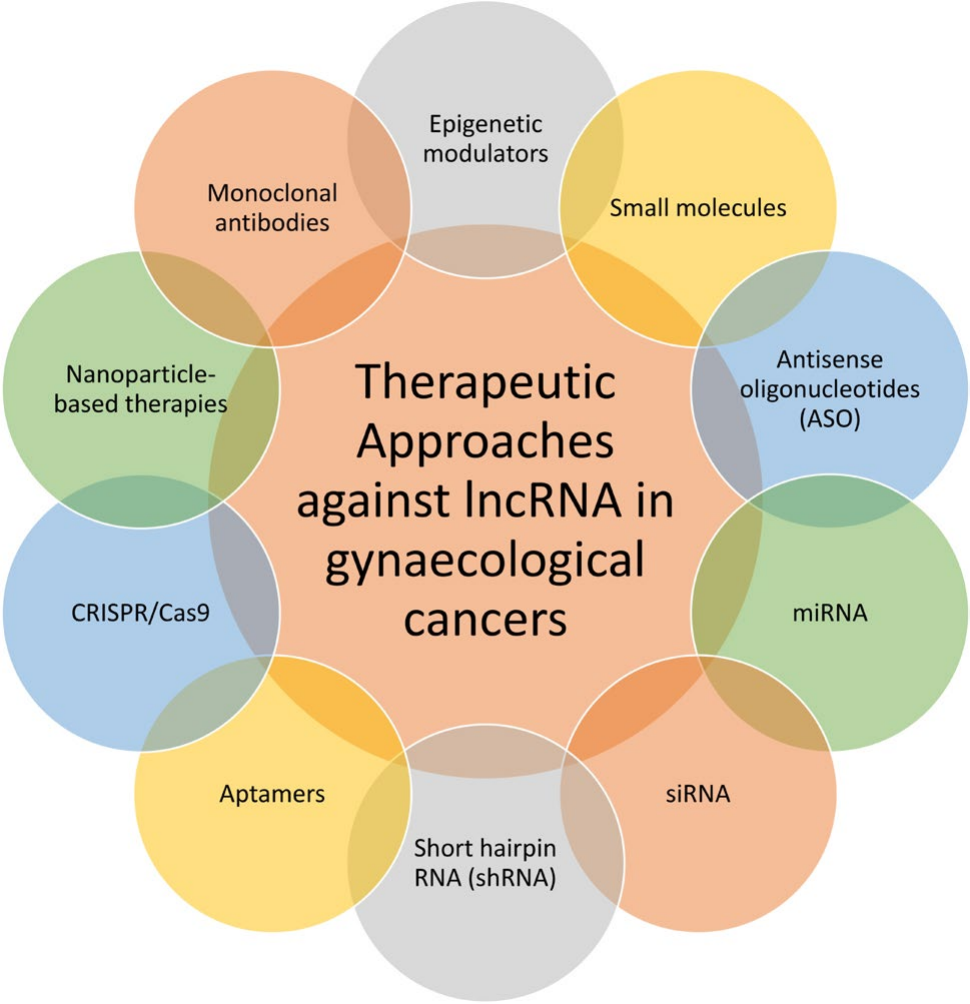
Therapeutic approaches against lncRNA. The general approaches that can be adopted to target lncRNAs therapeutically to treat cancer have been shown

Long non-coding RNAs have emerged as potential therapeutic targets for cancer, as they are able to influence extensive gene expression regulation and cellular processes. Antisense oligonucleotides (ASOs) are short, synthetic RNA or DNA molecules that can specifically target and bind to complementary lncRNA sequences, thereby blocking their function or promoting their degradation [35]. RNA interference (RNAi) is a process in which small RNA molecules, such as short interfering RNAs (siRNAs) or microRNAs (miRNAs), can be used to silence lncRNA expression [35]. siRNA-mediated knockdown of the lncRNA NCK1-AS1 has been shown to inhibit cervical cancer cell proliferation and invasion [36]. Antisense oligonucleotides (ASOs) can bind to lncRNA transcripts and induce their degradation or alter their splicing, leading to inhibition of their function. The CRISPR/Cas9 system can be used to target and disrupt specific lncRNAs by inducing site-specific DNA cleavage and subsequent degradation of the lncRNA transcripts. RNA aptamers are small, single-stranded RNA molecules that can bind to specific lncRNAs with high affinity and specificity, leading to inhibition of their function. Small molecule inhibitors can be designed to target specific lncRNA-protein interactions, thereby modulating lncRNA function [35]. For example, a small molecule inhibitor of the lncRNA HOTAIR has been shown to inhibit cervical cancer [37]. Barczak et al. showed that presenting the peptides derived from lncRNAs induces an immune response and lowers tumour development in mice models [38]. The development of lnc-RNA-based vaccines offers yet another novel therapeutic strategy. The therapeutic approaches being adopted to modulate lncRNA to target cancer cells has been shown in Fig. 3. Specific recent examples of therapeutic approaches against lncRNAs in gynaecological cancer has been discussed in the subsequent sections.

## Role of LncRNA in breast cancer

### Role in carcinogenesis

Based on the expression pattern breast cancer is categorized into four subtypes i.e., progesterone receptor (PR), human epidermal growth factor receptor 2 (HER2), estrogen receptor (ER). These subtypes include the basal-like, HER2-enriched, and luminal types A and B and triple-negative breast cancer (TNBC). The most prevalent breast cancer subtype, Luminal A, is distinguished by ER + and/or PR + /HER2 status, low-grade tumor, and favorable prognosis. About 10% of all breast malignancies is Luminal B-like (HER2 positive) is defined by ER + and HER2 overexpression or [39].

LncRNAs play a critical role in the regulation of gene expression and various biological processes in mammals. Recent research have confirmed with a lot of compelling evidence for the dysregulation of lncRNA expression in breast cancer, resulting in altered propagation of cell, cell death, migration, invasion, stemness, and drug resistance [39, 40]. Cell proliferation is a crucial factor in cancer progression, induced by aberrantly activated signaling pathways. Several lncRNAs have been identified as positive regulators of breast cancer cell proliferation, including DANCR, PVT1, CCAT1, KCNQ1OT1 and SPRY4-IT1 [41–44]. For instance, SPRY4-IT1 expression is linked with both size and pathological stages of the tumor in breast cancer patients, [39]. Tables 1 demonstrating the detail about the type of different types LncRNA, their expression, target, mechanism of action associated with breast cancer. DANCR and PVT1 are other lncRNAs implicated in promoting breast cancer cell proliferation through specific signaling pathways. on the other hand, PVT1, induces tumorigenesis and growth in TNBC by binding and increasing the stability KLF5 (Kruppel-like factor 5) via BAP1 resulting to overactivated β-catenin pathway [43, 44]. Additionally, some lncRNAs can modulate cancer cell proliferation by targeting miRNAs. For example, high expression of CCAT1 in TNBC tissues, downregulates miR-218 expression, subsequently affecting the downstream target ZFX, and reversing the miR-218 tumor-regressive effect [41]. Similarly, in vivo condition, KCNQ1OT1 modulate the miR-145/CCNE2 pathway to promote proliferation of tumor cells by acting as a competitive sponge for miR-145 [42]. Conversely, several lncRNAs exert tumor-suppressive effects on breast cancer cell proliferation. such as interaction of FOXO3 protein with LINC01355 stabilizes FOXO3 and in turn inhibits CCND1 transcription and hence breast cancer growth. Reduction of FOXO3 or overexpression of CCND1 can reverse the inhibitory action posed by LINC01355 on proliferation of breast cancer cells [45] In TNBC another tumor suppressor lncRNA (SONE) has been investigated. Reduced expression of SONE promote proliferation in TNBC cells. Additionally, in breast cancer tissue lncRNA MAGI2-AS3 (tumor suppressor) was found to be downregulated. MAGI2-AS3 or MAGI2 by blocking the Wnt/β-catenin pathway that deters proliferation and mitigates migration [46]. Furthermore, RNA-RNA interactions may regulate proliferation of breast cancer cell. Lon non-coding RNA PTCSC3, is reported to be negatively regulated by elevated expression of imprinted lncRNA H19 [47–49]. PTCSC3 inhibits TNBC cell proliferation by downregulating H19 expression, while H19 has no effect on PTCSC3 expression [48]. Another important tumor suppressor lncRNA is 9792 which blocks proliferative, invasive and metastatic capabilities of MDA-MB-231 and MDA-MB-468 cell lines (breast cancer) through ceRNA regulatory network.

**Table 1:** Role of lncRNA in breast  cancer

LncRNA	Expression	Target	Effect	Reference
DANCR	Upregulated	RXRA, AKT/PI3K signaling	Induces cell proliferation by promoting AKT/PI3K signaling via GSK-3β phosphorylation	[43, 44]
PVT1	Upregulated	KLF5, β-catenin signaling	Induces tumorigenesis and growth in TNBC by stabilizing KLF5 and activating β-catenin via BAP1	(Tang et al., [43, 44])
CCAT1	Upregulated	miR-218, ZFX	Amplifies proliferation by downregulating miR-218 and influences ZFX expression	Han et al., [41])
KCNQ1OT1	Upregulated	miR-145/CCNE2 pathway	Acts as a competitive sponge for miR-14 thus inducing tumor cell proliferation	(Feng et al., [42])
LINC01355	Downregulated	FOXO3, CCND1	Stabilizes FOXO3 and reduces CCND1 transcription thus inhibiting cell proliferation	(Ai et al., [45])
SONE	Downregulated	p53 (TP53), c-Myc	Regulates proliferation by altering miRNA expression, c-myc and p53 levels in TNBC	(Xu et al., [46])
H19	Upregulated	PTCSC3	Induces TNBC proliferation; negatively regulates PTCSC3 expression	(Raveh et al., [49])
BC069792	Downregulated	miR-658/miR-4739-KCNQ-JAK2-AKT network	Inhibits proliferation, invasion, and metastasis via the ceRNA network	(Y Zhang, [193])
NEAT1	Upregulated	miR-448, ZEB1	Promotes invasion and migration through miR-448 by acting as a sponge thereby regulating ZEB1	(Zhao et al., [52])
HOTAIR	Upregulated	PRC2, LSD1/CoREST complex	Promotes metastasis by acting as a scaffold for epigenetic protein complexes	(Tsai et al., [59])
MAGI2-AS3	Downregulated	Wnt/β-catenin pathway	Blocks Wnt/β-catenin pathway that deters proliferation and inhibits migration	(Xu et al., [46])
ATB	Upregulated	miR-200, restores Twist1 expression, IL-11	Promotes metastasis acting as a sponging miR-200, stabilizes IL-11 mRNA which activates STAT3	(Li et al., [56])
LINC00628	Downregulated	Bax, Bcl-2, caspase-3	Promotes apoptosis and suppresses proliferation by modulating gene expressions	Zhang et al., [68])
MALAT1	Upregulated	DBC1, p53 acetylation	Deters apoptosis by modulating p53 acetylation	(Chen et al., [69])
LINC00617	Upregulated	SOX2	Augments stemness and key role in EMT regulation	(Li et al., [87])
XIST	Downregulated	c-Met, EMT-related markers	Facilitates brain metastasis by stabilizing c-Met and promoting EMT	Xing et al., [65])
PICART1	Downregulated	AKT/GSK-3β/β-catenin signaling	Induces apoptosis and inhibits proliferation	(Cao et al., [67])
lncRNA-Hedgehog	Upregulated	GAS1, GLI1, OCT4, SOX2	Activates Hedgehog signaling, upregulating CSC-related gene expression	(Zhou et al., [60])
LINC-MAF-4	Downregulated	MAF	Regulates T-cell differentiation by recruiting chromatin moderators preventing MAF transcription	(Ranzani et al., [71])
lncDC	Upregulated	STAT3, SHP1	Promotes dendritic cell differentiation by enhancing STAT3 phosphorylation	(Wang et al., [73])
RP11-323N12.5	Upregulated	YAP1, Hippo signaling	Induces T-cell differentiation via Hippo signaling activation	(Wang et al., [72])

BC069792 can inhibit the proliferation, invasion and metastasis of breast cancer cells in vivo and in vitro through the ceRNA regulatory network of BC069792-hsa-miR-658/miR-4739-KCNQ-JAK2-AKT.Inc RNA BC069792 is expressed in both the cytoplasm and nucleus, which acts as a ceRNA sponge adsorbing miR-658 and miR-4739 and upregulates the transmembrane protein KCNQ4 expression, thereby inhibiting AKT phosphorylation and inhibiting the proliferation and metastasis of breast cancer (add citation).

Tumor invasion and metastasis are critical features of breast cancer and are leading causes of patient mortality. Aberrant expression of different lncRNAs contributes to an aggressive characteristic such as migration and invasion through various molecular mechanistics. EMT (Epithelial-mesenchymal transition) occurs when mesenchymal markers such as Twist1, N-cadherin, vimentin, fibronectin, and ZEB1 expression are upregulated while claudins, E-cadherin, and α-catenin, the epithelial junction proteins expression of are reduced [40, 50]. Research has established lncRNAs can regulate metastasis and invasion of tumor cells through interference with miRNAs [51]. For instance, lncRNA NEAT1, acts as ceRNA sponge for miR-448 to promotes migration and invasion, thereby regulating ZEB1. NEAT1 is also reported to negatively regulate miR-218, leading to enhanced breast cancer cell invasion [52]. Inhibition of NEAT1 has been shown to suppress the EMT via miR-211/ HMGA2 axis [53, 54]. NEAT1 influences several genes such as WNT4, ZEB1, TPD52, TIMM17A, KLF12, STAT3, FOXA1, HMGA2, ZFX, RRM2, EZH2, CPT1A, CCND1, FUS, Breast, TJP3, HuR, BCL2, ROCK1, ZEB1, BZW1, FGF9/VEGF, MEST, EZH2, Wnt3a/HMGA1, and let-7/ATGL through the miRNAs miR-129-5p, miR-448, miR-218-5p, miR-133b, miR-141-3p, miR-124, miR-23a-3p, miR-211, miR-138-5p, miR-21, miR-101, miR 107, miR-410-3p, miR-548ar-3p, miR-204, miR-146b-5p, miR-218, miR-1321, miR-124-3p, miR-34a-5p, miR-382-3p, miR-194, miR-4500, miR-365, miR-506, miR-124-3p, miR-144-3p, miR-146b-5p in various gynaecological cancers including ovarian, endometrium, cervical and breast cancer [55].

Similarly, suppression of vimentin and ZEB1 occurred when lncRNA TUSC8 competed with miR-190b-5p, that consequently led to the inhibition of metastasis in breast cancer [53]. Another overexpressed in breast cancer lncRNA is ROR that induces EMT by inhibiting miR-205 activity, hence preventing degradation of genes such as N-cadherin, vimentin, and ZEB1. This leads to lung metastasis in breast cancer and ROR Knockdown weaken this metastasis in in vivo condition. Additionally, inhibition of G9A methyltransferase (chromatin regulators) recruitment by ROR disrupts tescalcin (TESC) promoter histone H3K9 modification, resulting in abnormal breast cancer metastasis [54]. The lncRNA ATB is linked with augmented metastasis of lymph node and shorter overall survival in breast cancer patients. ATB functions as a miR-200 family sponge, restoring expression of Twist1 and hence promoting metastasis [56]. Moreover, ATB in breast cancer cell also enhances stemness and invasiveness through activating STAT3 the STAT3 pathway [57].

LINC01638 is a lncRNA that maintains the mesenchymal properties of TNBC cells. The inhibition of tumor growth and metastasis by LINC01638 knockdown have been established by both in vitro and in vivo studies [58]. The SPOP-mediated ubiquitination and degradation of c-Myc is prohibited by LINC01638 through its interaction with c-Myc. Twist1 levels are increased because of the c-Myc mediated transcriptional activation of metadherin (MTDH) expression, which facilitates TNBC invasion and metastasis [58]. Another lncRNA that is important for the spread of breast cancer is called HOTAIR. Two epigenetic protein complexes that encourage cancer metastasis use HOTAIR as a scaffold. The 3′ domain and 5′ domain of HOTAIR binds to the LSD1/CoREST/REST and PRC2 (polycomb inhibitory complex 2) respectively [59]. Microarray study demonstrates that the enhanced expression of genes regulated by HOTAIR (including STAT3, CD44, ZEB1, ALDH2, and vimentin) is associated with EMT and stemness [39].

Cancer stem cells (CSCs) are specialized cells with unimpeded capacity of proliferation self-renewal [60]. EMT (a process essential for the metastatic spread of cancer cells) stemness of cancer cells are directly related. Different signaling mechanisms, including the Wnt/$\beta$-catenin, Notch, and TGF- $\beta$ signaling pathways, regulate both CSC stemness and EMT [61, 62]. Additionally, EMT transcription factors including SLUG, ZEB1, and TWIST control the stemness genes in CSCs [63]. This suggests that EMT could serve as the foundation for CSC's stemness maintenance where LncRNAs have an integral role that helps in controlling the acquisition and maintenance of stemness in CSCs [62]. LncRNA Hedgehog (Hh) activates Hh signaling, by and upregulates the GL I1, OCT4 and SOX2 (CSC-related pluripotency genes) expression [60]. Therefore, inhibition of activated Shh-GLI1 signaling can lower OCT4 and SOX2 levels. Additionally, silencing of lncRNA-Hh in Twist-positive breast cancer cells suppressed the activated GL I1 signaling that ultimately reduced the efficiency of mammosphere formation and carcinogenesis of transplanted tumor cells and hence can be as prognostic and diagnostic molecules [60]

Another lncRNA, LINC00617, promotes invasion and metastasis in breast cancer by the enhancing expression of CD44 and lack of CD24 markers. LINC00617 achieves these effects by upregulating SOX2 expression, a key transcription factor involved in EMT regulation [64]. The lncRNA XIST exhibits a negative correlation with brain metastasis in breast cancer patients. Reduced XIST expression, through moesin (MSN)-mediated protein stabilization, promotes EMT through and the c-Met activation. This activation promotes tumor cells stemness and contributes to their invasive properties. Studies using mouse models have shown in mammary glands XIST knockout boosts the primary tumor growth and brain migration and invasion, further emphasizing its role in development of breast cancer [65]. Collectively, these findings highlight the importance of lncRNAs, such as LINC00617 and XIST, in regulating EMT, stemness, and the invasion and migratory abilities. Understanding the intricate mechanisms involving lncRNAs in breast cancer metastasis can provide valuable insights for developing novel diagnostic and therapeutic strategies to combat this devastating disease [64, 65].

LncRNAs also acts as a critical regulators of cell apoptosis, through balancing of pro-apoptotic and anti-apoptotic gene expression, which is frequently disrupted in cancer, leading to uncontrolled cell proliferation [66]. The prominent lncRNA is PICART1 (p53-inducible cancer-associated RNA transcript 1, a tumor suppressor) which promotes apoptosis via AKT/GSK-3β/β-catenin signaling pathway [67]. Further, lncRNAs can modulate apoptosis via the caspase signaling pathway. Similarly, LINC00628, facilitates apoptosis through modulating Bax, Bcl-2 and caspase-3, expression [68]. Less overall survival rate in breast cancer patients have been reported with reduced manifestation of LINC00628 along with poor prognosis. Conversely, increased expression of LINC00628 has been shown to impede growth, migration and invasion, and arresting cell cycle at G0/G1 phase [68].

Another lncRNA, MALAT1, has been shown to interact with DBC1 to modulate p53 acetylation, a modification that inhibits apoptosis [69]. Similarly, overexpression of lincRNA-APOC1P1-3 through promoter hypomethylation is linked to magnitude of the tumor. Moreover, APOC1P1-3 can suppress apoptosis through direct binding and reducing the a-tubulin acetylation, thus deactivating caspase-3. Manipulation of lncRNA to regulate apoptosis in transformed cell holds promise as an effective strategy for treatment [68].

Recently research focus on the key role played by lncRNAs in regulating the cancer immune response as they play a very substantial role in the development of carcinogenesis [39, 70]. Although immune proteins are not encoded by lncRNAs but they influence immune cell function by inducing T cell differentiation, polarizing macrophages, and impacting dendritic cell antigen presentation abilities. Cancer immunotherapy, which harnesses the power of the immune system to eliminate cancer cells, has emerged as a promising treatment option. However, immunosuppression often occurs due to factors such as loss of antigenicity, oncogenic signals reactivation, evasion of immune checkpoint, and increased AICD (T lymphocyte activation-induced cell death) during immunotherapy [39].

Comprehensive research has been carried out by different researcher on T lymphocytes and lncRNAs. It was demonstrated that the MAF’s expression is negatively linked with linc-MAF-4, a chromatin-related T helper (Th)1-specific lincRNA. LSD1 and EZH2 are enlisted by linc-MAF-4 to act as chromatin moderators, which prevent MAF transcription. By increasing MAF, linc-MAF-4 downregulation promotes CD4 + [71]. In addition, T cells can take up the tumor-derived exosomes carrying lncRNAs which then can induce the differentiation of regulatory T cells (Tregs). For instance, exosomes rich in the lncRNA RP11-323N12.5 are secreted by tumor cells and picked up by T cells [72]. The lncRNA RP11-323N12.5 causes T cells to develop into Tregs by inducing the Hippo signaling pathway that subsequently activates YAP1 in T lymphocytes. In addition, tumor-derived exosomes carrying lncRNAs can be picked up by T lymphocytes to induce the differentiation of regulatory T cells (Tregs). For instance, tumor cells secrete the exosomes rich in the lncRNA RP11-323N12.5 which is then picked up by T lymphocytes which. causes T cells to develop into Tregs by inducing the Hippo signaling pathway [72]. DCs influence both innate and adaptive immune responses since they are the primary APC in the mammalian immune system. LncRNA controls how DC is activated as well. For instance, the differentiation of DC is associated with a DC-specific lncRNA (lncDC). LncDC can direct the differentiation of DC, through preventing STAT3 and SHP1 from combining to phosphorylate STAT3 [73]. Loss of lnc DCs lowers the capacity of DCs to activate T cells and affects DC development both in vitro and in vivo [73]. Breast cancer malignancy and metastasis are significantly influenced by phenotypic shift in TAMs [74]. LncRNA are also directly or indirectly involved in macrophage polarization and it was revealed that in M1-type macrophages, XIST lncRNA was elevated which are pro-inflammatory. By preventing the production of C/EBPa and KLF6, XIST knockdown in M1 can cause M1 to change into an anti-inflammatory M2 macrophage (M2), which in turn encourages the propagation and migration of tumor cells [75]. While lncRNA XIST directly stimulates M2 polarization of macrophages, another lncRNA produced indirectly does so by causing the release of cytokines by tumor cells. For instance, breast cancer cells that overexpress the linc00514 gene have a higher proportion of macrophages that are positive for the M2 markers CD206 and CD163. Linc00514 increases the STAT3 phosphorylation to increase Jagged1expression, which in turn activates the Notch signaling pathway. Next the activated Notch pathway encourages the release of IL-4 and IL-6 from breast cancer cells which cause macrophage M2 polarization [76].

In summary, aggressive phenotype in breast cancer cells is caused by dysregulated expression of lncRNAs. These lncRNAs modulate various molecular pathways involved in EMT, miRNA regulation, and autocrine signaling, ultimately promoting the invasive behavior. Therefore, understanding the functional mechanisms of these lncRNAs provides valuable insights for the development of therapeutic strategies.

In recent years, advancements in transcriptome profiling technology have expanded our understanding of the diverse and complex compositions of human RNA molecules. Among them, lncRNAs are documented to be crucial players in normal physiological development, and their dysregulated expression is closely associated with breast cancer. Thus, lncRNAs hold promise as biomarkers for tumor diagnosis, prognostication, and prediction of disease development [39].

In the realm of improving early diagnosis of breast cancer and clinical effectiveness, the identification of biomarkers and molecular subtypes is of utmost importance. Clinical data have shown that certain lncRNAs exhibit significant aberrant expression in breast cancer lesions and precancerous tissues. For instance, lncRNAs UCA1, HIF1A-AS2, and ANRIL, are notably overexpressed in TNBC patients plasma compared to individuals without TNBC, suggesting their potential as diagnostic biomarkers specific to TNBC [77]. Additionally, in TNBC, it has been revealed that hypermethylation of LINC00299 in the peripheral blood serve as a valuable circulating biomarker hence offering excellent diagnostic value [77].

Recently a study highlighted that androgen receptor (AR) expression in a subtype of TNBC, is linked with upregulated expression of HOTAIR and the LAR (luminal androgen receptor)253. Fragments derived from circulating HOTAIR are detectable in the serum of both healthy individuals and breast cancer patients, signifying its role as a biomarker for breast tumor [39, 78].

Furthermore, the lncRNA CCTA1 has been found to promote β -catenin translocation via interacting with ANXA2 and miR-148a/152 and CCTA1 also enhances TCF4 expression though competitively binding to miR-204/211. On the other hand, TCF4 can promote CCAT1's transcription through binding at the promoter region of the lncRNA CCAT1. This reciprocal regulation creates a positive-feedback circuit in breast cancer including CCAT1, TCF4, and CCAT1, underscoring the significant role of CCAT1 in disease development and its potential as a target for significance of lncRNAs for the early diagnosis of breast cancer, presenting promising applications in clinical practice. By utilizing lncRNAs as diagnostic tools, the field aims to enhance early detection and improve patient outcomes in breast cancer management [79].

Despite significant advancements in breast cancer treatment, the prognosis for patients remains poor due to frequent distal metastases and chemotherapy resistance. Aberrant expression of distinct lncRNA serves as potential prognostic biomarkers in breast cancer patients. Notably, in plasma (lncRNAs such as HISLA, HOTAIR, GAS5 and H19), as well as in tissues (MALAT1, LINC000473, LINP1, and TINCR,) have shown correlations with unfavorable prognosis [80]. HOTAIR has been established as a diagnostic biomarker and is associated with reduced survival time of the patient. Kaplan–Meier survival analysis has revealed negative correlation between the disease-free survival (DFS) with amount of circulating HOTAIR in plasma, among patients [80]. Similar correlations have been observed for lncRNA in plasma (such as GAS5, H19, HISLA) and in tissue (MALAT1), where elevated levels promote metastasis of the lymph node (LNM) with the reduced overall survival (OS). Furthermore, preoperative and postoperative analysis of plasma samples has shown that levels of GAS5, H19, and HISLA was reduced after surgery which are indicative of a better prognosis [39]. MALAT1 is an important pro-inflammatory factor and higher expression predicts shorter DFS (5 year). Another important lncRNAs are LINP1, TINCR and LINC000473 whose overexpression is also correlated with advanced tumor stage, LNM, and poor pathological differentiation. Patients with high LINP1 expression exhibit shorter OS and DFS compared to those with low LINP1 expression [81, 82] (Table 1).

Recently several novel lncRNAs have been identified in TNBC patients that act as predictive biomarkers including ATB, PHACTR2-AS1, MIR503HG, NAMPT-AS, DANCR, and LINC01089. Similarly, in TNBC patients increased ATB and NAMPT-AS expression is inversely associated with OS and disease-free survival (DFS) [39, 56, 89]. In contrast, MIR503HG (a tumor suppressor) in TNBC serves as a prognostic marker, as its low expression being an independent poor prognostic factor for OS. LINC01089 and PHACTR2-AS1 exhibit similar prognostic roles [44]. These findings advocate that combining varying expression of lncRNAs with integrated mRNA-lncRNA signatures can serve as powerful biomarkers for clinical prognosis as their aberrant expression patterns provide valuable insights into disease progression and patient outcomes, allowing for better prognostication and personalized treatment strategies.

### Therapeutic strategies

The finding on the structural information and functional roles of lncRNAs has paved the way for the development of small molecule inhibitors targeting these molecules, holding great potential for therapeutic strategies. Significant progress has been made in the field of novel drugs that target lncRNAs (including phytochemicals and synthetic compounds). Various approaches have been developed including siRNAs, small molecule inhibitors, antisense oligonucleotides (ASOs), and CRISPR-Cas9. Additionally, indirect modulation of lncRNAs has also shown promising results in the direction of therapeutic development [39].

The exploration of lncRNAs as therapeutic targets has become a prominent area of interest in breast cancer prognosis, diagnosis, and treatment strategies. The shell of the cashew nut (Anacardium occidentale) is rich in phenolic lipids called anacardic acids. In breast cancer cell lines, a particular anacardic acid congener (AnAc 24:1n5) may suppress the expression of endogenous estrogen regulating genes such CTSD, CCND1, and TFF1. By altering the expression of several lncRNAs (including UBL7-AS1, CFLAR-AS1 and MIR210HG) in breast cancer cell lines (MCF-7 and MDA-MB-231), this phytochemical was found to enhanced PDK4 and reduced the expression of INSIG1, TGM2, and SCD gene [83].

Baicalein is a flavonoid with a repertoire of pharmacological properties and regulates the expression of tumor suppressive lncRNA in breast cancer. Breast cancer tumors exhibit downregulation of the PAX8-AS1-N lncRNA which results in poor survival of the patients [39]. siRNAs mediated reduced PAX8-AS1-N expression led to a higher cell proliferation and lowers apoptosis [90].

Bharangin (anticancer activity) is a phytochemical found in *Pigmacopremna herbacea* [91]. Bharangin was evidenced to mitigate breast cancer cell (MCF-7, MDA-MB-231, -453, and -468) lines from migrating, proliferating, and cell cycle passage from via regulating various lncRNAs [92] (Table 2).

Calycosin and genistein belong to isoflavones group with potent anticarcinogenic properties which can lower the risk of estrogen-correlated malignancies. Calycosin suppresses the proliferation by overactivating WDR7-7-GPR30 signaling [84, 86]. according. Additionally, the calycosin impacted inhibition was more profound on ER + breast ER-breast cancer cells. This result might be attributed to calycosin’s ability to upregulate RASD1 expression in ER + cells while downregulating ER and miR-375 [86, 93]. Calycosin and genistein in combination induced apoptosis in MCF-7 cells. Notable, calycosin was tested to be more effective than genistein in mitigating MCF-7 cells [84]**.**

Ginsenoside is a naturally occurring steroid glycoside byproduct that has a variety of chemical structures and pharmacological effects. Through epigenetic alterations, these substances change how lncRNAs and other cancer-related genes are expressed [85, 94]. Breast cancer cells overexpress lncRNA-C3orf67-AS1, and suppressing it in MCF-7 cells with a particular siRNA prevents colonization and proliferation by triggering death. Ginsenoside (Rh2) is crucial in the treatment of breast cancer since it has the ability to inhibit C3orf67-AS1 by inducing hypermethylation of the gene's promoter in MCF-7 cells [94]. Additionally, ginsenoside (Rg3) treatment of MCF-7 cells revealed that Rg3 was capable of upregulating and downregulating the expression of two oncogenic and non-oncogenic lncRNAs (RFX3-AS1 and STXBP5-AS1) via hypermethylation and hypomethylation, respectively [85, 86].

Another important lncRNAs, such as HOTAIR can be regulated by siRNAs which in turn prohibits proliferation and metastasis in breast cancer (Li et al. [64]). Similarly, nanoparticle-encapsulated siRNAs can target lncRNA BM which promotes breast cancer brain metastasis [88]. Similarly, an oncogenic lncRNA DANCR can be effectively downregulated by nanoparticle-mediated siDANCR that consequently thwarts proliferation and inhibits migration and invasion in TNBC cell lines [95].

ASOs, synthetic oligonucleotides that complementarily bind to target lncRNAs and can be cleaved by RNase H, have also shown promise [96]. For instance, ASOs targeting LINC02273 disrupt the hnRNPL-LINC02273 complex, which leads to decreased expression of AGR2, a gene associated with breast cancer metastasis. Both in vitro and in vivo inhibition of AGR2 expression effectively suppresses metastasis [97]. Moreover, ASOs targeting NRAD1 (LINC00284) have demonstrated enhanced apoptosis, reduced tumor proliferation, and the number of cancer cells that exhibit stemness property in TNBC tumors(Xiu et al. [97]; Lee and Mendell [96]) (Table 2).

The genome editing through CRISPR-Cas9 technique is powerful tool for knocking out lncRNAs, as supported by increasing evidence [98]. This technology enables precise deletion of specific genomic locations with high accuracy and fidelity. Researchers have utilized CRISPR-Cas9 to investigate the role of lncRNAs in cancer and their potential as therapeutic targets.

For example, NQO1 [NAD(P)H: quinone oxidoreductase 1] expression in radiation-resistant breast cancer cells, had been positively regulated by lncRNA NEAT1. By employing CRISPR-Cas9 to inhibit NEAT1 expression, the sensitivity to radiation of the radiation-resistant cells were found to be increased that resulted in reduced cell multiplication, expression of tumor stemness markers like SOX2, BMI1, and OCT4 [98, 99]. Another study focused on the lncRNA MEG3 and its knockout using the CRISPR-Cas9 system, which caused a substantial decrease in the invasive ability of TNBC cells [100]. Therefore, these findings highlight the potential of CRISPR-Cas9 as a precise method of genome editing to eliminate aberrantly expressed lncRNAs (oncogenic) in cancer cells, is a valuable approach towards the development of precision medicine therapies. The utilization of CRISPR-Cas9 in lncRNA research opens up exciting avenues for therapeutic interventions in cancer treatment.

**Table 2:** Therapeutic strategies involving lncRNA in Breast cancer

Therapeutic agent	Sample	Molecular target	Effect	Reference
Baicalein (phytochemical)	Breast cancer cell line	Enhanced PAX8-AS1-N lncRNA (tumor suppressive lncRNA)	Enhanced expression of CDKN1A, PTEN, and BTB that indues apoptosis and limits proliferation and by blocking miR-17-5p	(Jin et al. [39]), Yu et al. [90])
Anacardic acid (phenolic lipids)	MCF-7 and MDA-MB-231	Downregulate lncRNA UBL7-AS1, CFLAR-AS1 and MIR210HG	Suppress the expression of endogenous estrogen regulating genes such CTSD, CCND1, and TFF1	[83]
Bharangin (phytochemical)	MCF-7, MDA-MB-231, -453, and -468) lines	Regulating lncRNAs (MEG-3, H19, NEAT1, GAS-5, and MHRT) and suppressing NF-B activity	Mitigates migrating, proliferating, and cell cycle passage	(Gupta et al. [91]), (Awasthee et al. [92])
Calycosin (isoflavones)	ER + breast ER-breast cancer cells	Overactivating WDR7-7-GPR30, RASD1 expression in ER + cells while downregulating miR-375	Suppresses the proliferation	(Chen et al. [84]), (Kalhori et al. [86]), (Křížová et al. [93])
Calycosin and genistein	MCF-7 cells	Deactivate HOTAIR/p-Akt via decreasing the expression of p-Akt	Induces apoptosis, calycosin was found tested to be more effective than genistein	Teng et al. [85]; Jeong et al. [94]
Ginsenoside (steroid glycoside)	MCF-7 cells	Inhibit C3orf67-AS1 by inducing hypermethylation	Prevents colonization and proliferation by triggering death	[85, 86]
siRNAs	Breast cancer	Downregulate HOTAIR	Prohibits proliferation and metastasis	(Li et al. [87])
Nanoparticle-encapsulated siRNAs	MDA-MB-231, MCF7	Downregulate lncRNA BM	Promotes breast cancer brain metastasis	[88]
ASOs	MDA-MB-231 and BT549	Targets LINC02273 that disrupt the hnRNPL-LINC02273 and leads to inhibition of AGR2 expression	Suppresses metastasis	Xiu et al. [97];
	TNBC	Targets LINC00284	Enhances apoptosis, reduces proliferation, stemness property of TNBC tumor cells	Lee and Mendell [96]

## Role of lncRNA in cervical cancer

### Role in carcinogenesis

Cervical cancer is one of the most common cancers affecting women worldwide, with human papillomavirus (HPV) infection being the major risk factor for the development of this cancer. Several lncRNAs such as H19, LINP1, HOTAIR, CCAT1, CDKN2B-AS1, NEAT1, PVT1, TMPOP2, TUG1, UCA1, XIST are upregulated in cervical cancer and is associated with poor prognosis and tumour progression. HOTAIR plays an oncogenic role in cervical cancer by promoting cell proliferation, migration, invasion and autophagy, inhibiting cell apoptosis, stimulating angiogenesis, accelerating cell cycle progression, and inducing epithelial-mesenchymal transition [31] lncRNA HOTAIR is upregulated in cervical cancer tissues and regulates the expression of several genes including Wnt/β-catenin pathway, p21, several miRNA such as miR206, miR-143-3p, miR-331-3p, miR-148a [15, 101, 102]. It also has been shown to modulate several HPV genes such as E7 and influences cell proliferation, and migration [17]. UCA1, is also upregulated in cervical cancer and promotes the upregulation of the glycolytic pathway and can regulate radio-resistance [103]. XIST upregulation enhances cell proliferation and cell cycle [104]. PVT1 promotes cancer proliferation, cell cycle progression and migration via the EZH2 epigenetic route [105]. Another study also showed that upregulation of PVT1 mitigates proliferation and invasion of cervical cancer cells. H19 upregulation also modulates SIRT1 expression [106].

Several lncRNAs MEG3, TUSC8, CRNDE, have been shown to act as tumour suppressors in cervical cancer. GAS5 is downregulated in cervical cancer tissues and cell lines and inhibits cell proliferation and invasion by regulating the expression of target genes such as MMP2 and MMP9 and impacts the STAT-3 pathway [21]. MALAT1, is typically downregulated in cervical cancer and impacts cell viability, cell migration and invasion, via modulating miRNA [107]. GAS5 lncRNA is downregulated in cervical cancer and thereby induces apoptosis, prevent invasion, promote chemo-sensitivity to cisplatin, downregulate PI3K/AKT signalling via miR-21 and PTEN route [20]. *PTEN* and *FOXO1* is quenched by upregulation of GAS5 [108].

Several studies have identified lncRNAs that are induced by HPV infection and play important roles in the pathogenesis of cervical cancer. Liu et al. have noted that following HPV infection, 194 lncRNAs are differentially regulated, one of the important ones being lnc-FANCI-2 [109]. lncRNA ANRIL has also been shown to modulate miR-186 and promote cervical cancer [110]. Its upregulation is associated with metastasis, disease severity and poor prognosis and its high expression can be deemed as a prognostic indicator [111, 112]

Several studies have reported the differential expression of lncRNAs in cervical cancer tissue compared to normal cervical tissue, as well as their correlation with clinicopathological features of cervical cancer, including tumour stage, grade, and lymph node metastasis. lncRNAs that have been investigated as potential biomarkers for cervical cancer include HOTAIR MALAT1, HIF1A-AS2, PRNCR1 and UCA1 [16, 19, 35, 111]. lncRNA PCAT6 modulates the Wnt- β-catenin pathway and has been labelled as a good candidate for prognostic indicator [32]. Another recent study has identified a 15-lncRNA expression signature that can be used as a prognostic indicator and to predict patient outcome. This signature includes BAIAP2-AS1, RP11-203J24.8, LINC01133, RP1-7G5.6, RP11-147L13.15, SERHL, CTC-537E7.3, RP11-440L14.1, RP11-131N11.4, ILF3-AS1, RP11-80H18.4, RP11-1096G20.5, CTD-2192J16.26, RP11-621L6.3, and RP11-571M6.18 [30]. Liu et al. used the expression profile of six lncRNAs, including UBL7-AS1, LIPE-AS1, PCED1B-AS1, and NCK1-DT which are upregulated in cervical cancer cells and influence the immune moieties to build a prognostic signature [113] (Table 3).

**Table 3:** Role of lncRNA in cervical cancer

lncRNa	Expression	Target	Effect	References
HOTAIR	Upregulated	Regulates Wnt/β-catenin pathway, p21, miR206, miR-143-3p, miR-331-3p, miR-148a HPV genes E7	Promotes cell proliferation, migration, invasion, autophagy, inhibits cell apoptosis, stimulates angiogenesis	[31] [15, 101, 102]. (Sharma et al. [17])
UCA1	Upregulated	upregulation of the glycolytic pathway	Regulate radio-resistance	Fan et al. [107])
XIST	Upregulation		Enhances cell proliferation and cell cycle	(Zhu et al. [104])
PVT1		EZH2 epigenetic	Cancer proliferation, cell cycle progression and migration	(Zhang et al. [105])
H19		SIRT1 expression		(Ou et al. [106])
GAS5	Downregulated	MMP2 and MMP9 and impacts the STAT-3 pathway downregulate PI3K/AKT signalling via miR-21 and PTEN route PTEN and FOXO1 is quenched	And inhibits cell proliferation and invasion induces apoptosis, prevent invasion, promote chemo-sensitivity to cisplatin	(Zhang and Gao [21])
MALAT1	Downregulated	miRNA	Cell viability, cell migration and invasion,	(Han et al. [107])
ANRIL		miR-186	Metastasis, disease severity and poor prognosis	(Zhang et al. [101])(Zhang et al. [23]; Yao et al. [111])

### Therapeutic strategies

Paclitaxel inhibits the progression of cervical cancer by inhibiting autophagy via lncRNARP11-381N20.2 [114]. A phytochemical, baicalein was found to lower the expression of lncRNA BDLNR, downregulated PI3K/Akt pathway leading to inhibition of tumour growth, migration and proliferation in cervical cancer cells and mice models [115]. Artesunate, a derivative isolated from artemisinin compound down-regulates HOTAIR expression and consequently reduces the number of metastatic nodules in female athymic nude mice bearing HeLa cells [116]. Propofol, an anaesthetic has been shown by several studies to induce apoptosis by specifically downregulating HOTAIR expression and its associated pathways like mTOR pathway, in various cervical cancer cells like HeLa, SiHa as well as in mice models [37, 117]. Metformin has also been shown to have anti-cancer effect on cervical cancer cells both in vitro and in animal models by acting on the MALAT1/miR-142-3p sponge action [118]. The stability of lncRNA GAS5 has been shown to be increased by the action of its anti-sense moiety GAS5-AS1 leading to anti-cancer effect [119]. Cervical cancer cells treated with lidocaine showed marked reduction in proliferation due to the effect on the lncRNA-MEG3/miR-421/BTG1 signalling pathway [120].

MALAT1 siRNA has been shown to reduce colony formation as well as increase apoptosis in cervical cancer cell lines (CaSki and Hela) [121]. Knockdown of UCA1 using siRNA-based targeting was found to reverse radioresistance of cervical cancer cell lines (SiHa and HeLa) [103]. lncRNA NEAT1 knock-down was able to significantly reduce LDHA expression, lower the rate of glycolysis and sensitize the cervical cancer cells to 5-Fluorouracil [122]. Silencing of lncRNA NCK1‑AS1 impedes proliferation of cervical cancer cells and impairs miRNA activity [36]. siRNA/shRNA-mediated silencing of the lncRNA MALAT1 results in tumour progression, cell motility, and viability along with apoptosis in adenocarcinoma, cervical cancer. A strong anti-cancer response via the miR-642a-5p/LGMN axis is observed in cervical cancer cells where the lncRNA PCGEM1 has been knocked down [123] (Table 4). Oncogenic ZFAIS can be suppressed to reduce the proliferative, migratory and invasive capability by using siRNA treatment of cervical cancer cells in mice model. ZFAS1 siRNA therapeutic also increased the sensitivity to cisplatin in cervical cancer [31, 124].

**Table 4:** Therapeutic strategies involving lncRNA in cervical cancer

Therapeutic agent	Sample	Molecular target	Effect	Reference
Baicalein (Phytochemical)	Cervical cancer cells and mice models	Downregulate lncRNA BDLNR and PI3K/Akt pathway	Inhibition of proliferation migration and tumour growth	(Yu et al. [115])
Paclitaxel	Cervical cancer cells	Via lncRNARP11-381N20.2	By inhibiting autophagy	(Zou et al. [114])
Artesunate	Female athymic nude mice bearing HeLa cells	Down-regulates HOTAIR	Reduces the number of metastatic nodules	(Zhang et al. [11])
Propofol	Cervical cancer cells like HeLa, SiHa as well as in mice models	Downregulating HOTAIR and mTOR pathway	Induce apoptosis	(Zhang et al. [37]; Li et al. [64])
Metformin	In vitro and in animal models	MALAT1/miR-142-3p sponge action	Anti-cancer effect	(Xia et al. [118])
Lidocaine		lncRNA-MEG3/miR-421/BTG1 signalling pathway	Reduction in proliferation	(Zhu and Han [120])
MALAT1 siRNA	Cervical cancer cell lines (CaSki and Hela)	MALAT1	Reduce colony formation as well as increase apoptosis	(Lu et al. [121])
siRNA-based targeting of UCA1	Cervical cancer cell lines (SiHa and HeLa)	Knockdown of UCA1	Reverse radioresistance	(Fan et al. [103])
lncRNA NEAT1 knock-down		lncRNA NEAT1	Reduce LDHA expression, lower the rate of glycolysis and sensitize the cervical cancer cells to 5-Fluorouracil	(Shao et al. [122])
Silencing of lncRNA NCK1‑AS1		miRNA activity	Impedes proliferation of cervical cancer cells and impairs miRNA activity	(Huang et al. [36])

## Role of lncRNA in ovarian cancer

### Role in carcinogenesis

Ovarian cancer is a common gynaecological malignancy, affecting the ovaries and is burdened by late detection as well as a high mortality rate. Over 300000 new cases, and more than 207,000 women succumbed to ovarian cancer worldwide in 2020 alone [125].

In ovarian cancer, it has been shown that the lncRNAs HOTAIR, MALAT1, LINC01127, AB073614, ElncRNA1 and ANRIL modulate the PI3K pathways, while LncRNA HOST2 GAS5, TUBA4B modulate the RAS pathway and lncRNAs TUG1, HOXD-AS1, SNHG20 and EBIC modulate the Wnt/β-catenin pathway (reviewed in [126]). The lncRNA GAS5 sponges miR21, alters PARP1 expression modulating cell proliferation in ovarian cancer cells [127, 128]. Another example is that of LINC00092 that has been shown to promote CAF-induced cancer progression and increase glycolysis in the cells [129]. lncRNA UCA1 upregulation also contributes to the evasion of apoptosis, increase in cell viability and promotion of drug resistance [130]. MALAT1, often upregulated in other cancers is also found to be overexpressed in ovarian cancer cells. It up-regulates SOX2, downregulates miR-133a and promotes the development of all hallmarks of carcinogenesis [131, 132]. A study also shows that lncRNA HULC binds to ATG7 and modulates autophagy components to induce carcinogenic changes in ovarian cancer cells. It has also been reported to upregulate the PI3K/AKT/mTOR signalling pathway [133]. lncRNA PVT1 is also frequently overexpressed and enables the proliferation and metastasis of ovarian cancer cells via modulation of miR133a [134]. Several lncRNAs like ZFAS1, MALAT1, H19 have been shown to increase resistance to cisplatin while lncRNA like GAS5, NEAT1 lower cisplatin resistance in ovarian cancer cells [135]. MIAT is overexpressed in ovarian cells, sponges the miR-150-5p and functions as an oncogene [136]. Likewise in ovarian cancer, lncRNA H19 modulates various genes and pathways and suppresses miR-612, miR-342-3p, miR-124-3p and miR-216a-5p modulating HOXA10, IER3, ITGB3 and ACTA2 and influencing endometrial cancer cell viability, invasion and overall survival. lncRNA H19 sponges 4miR-138-5p, increasing SIRT1 leading to inhibition of apoptosis and proliferation in cervical cancer [137]. It is also expected to serve as a key biomarker and therapeutic target. TUG1 is typically upregulated in ovarian cancer and inhibits the onset of apoptosis [138]. Further, several lncRNAs have been linked with p-53 in ovarian cancer, of which H19, MALAT1, PVT1 and LINC-ROR are upregulated while MEG3 and PANDA are known to be downregulated [139]. These lncRNAs in turn modulate various cancer hallmarks including proliferation, apoptosis, migration, EMT, cisplatin-resistance, DNA repair and are associated with recurrence risk. A study by Hu et al. indicates that the downregulation of lncRNa XIST in ovarian cells correlates with improved prognosis [140].

A prognostic signature with eleven lncRNAs, including NEAT1, HOTAIR, MALAT1, ANRIL, CCAT2, ZFAS1, UCA1 have been shown to predict poor patient survival and outcome in ovarian cancer patients [141]. A similar profile using the downregulation of five lncRNA and upregulation of eight lncRNA have been shown to be a poor prognosis indicator [139]. It is interesting to note that some lncRNAs such as HOTAIR, ANRIL, GAS5, NEAT1, CCAT2, UCA1 are consistently reported to be associated with several prognostic indicators such as advanced FIGO stage, increased tumour growth, lymph node metastasis and lowered survival measures [126] (Table 5).

**Table 5:** Role of lncRNA in ovarian cancer

lncRNA	Expression	Target	Effect	References
HOTAIR	Upregulated	PI3K pathways	Promotes tumour growth, metastasis, associated with poor prognosis	Zamaraev et al. [126]
MALAT1	Upregulated	PI3K pathways, SOX2, miR-133a	Promotes proliferation, migration, invasion, cisplatin resistance, and other hallmarks of carcinogenesis	Bai et al. [131]; Gordon et al. [132]
LINC01127	Upregulated	PI3K pathways	Impacts proliferation, migration, and invasion	Zamaraev et al. [126]
AB073614	Upregulated	PI3K pathways	Impacts proliferation, migration, and invasion	Zamaraev et al. [126]
ElncRNA1	Upregulated	PI3K pathways	Impacts proliferation, migration, and invasion	Zamaraev et al. [126]
ANRIL	Upregulated	PI3K pathways	Promotes tumour growth, metastasis, associated with poor prognosis	Zamaraev et al. [126]
HOST2	Upregulated	RAS pathway	Impacts tumour growth and progression	Zamaraev et al. [126]
GAS5	Downregulated	RAS pathway, miR-21, PARP1	Reduces proliferation, lowers cisplatin resistance	Li et al. [127]; Ma et al. [128]
TUBA4B	Downregulated	RAS pathway	Impacts tumour suppression and progression	Zamaraev et al. [126]
TUG1	Upregulated	Wnt/β-catenin pathway	Inhibits apoptosis, promotes tumour progression	Kuang et al. [138]
HOXD-AS1	Upregulated	Wnt/β-catenin pathway	Impacts tumour progression	Zamaraev et al. [126]
SNHG20	Upregulated	Wnt/β-catenin pathway	Impacts tumour progression	Zamaraev et al. [126]
EBIC	Upregulated	Wnt/β-catenin pathway	Impacts tumour progression	Zamaraev et al. [126]
LINC00092	Upregulated	Glycolysis pathways	Promotes CAF-induced cancer progression, increases glycolysis	Zhao et al. [129]
UCA1	Upregulated	Apoptosis regulators	Promotes drug resistance, increases cell viability, and evasion of apoptosis	Wang et al. [130]
HULC	Upregulated	ATG7, PI3K/AKT/mTOR pathway	Modulates autophagy, induces carcinogenic changes	Chen et al. [133]
PVT1	Upregulated	miR-133a	Promotes proliferation and metastasis	Yang et al. [181]
ZFAS1	Upregulated	Unknown	Increases cisplatin resistance	Elsayed et al. [135]
NEAT1	Downregulated	Unknown	Lowers cisplatin resistance	Elsayed et al. [135]
H19	Upregulated	p53 pathway	Increases cisplatin resistance, impacts tumour hallmarks	Luo et al. [139]
MEG3	Downregulated	p53 pathway	Promotes tumour suppression	Luo et al. [139]
PANDA	Downregulated	p53 pathway	Promotes tumour suppression	Luo et al. [139]
MIAT	Upregulated	miR-150-5p	Functions as an oncogene, key biomarker and therapeutic target	Zhou et al. [31]
XIST	Downregulated	Unknown	Correlates with improved prognosis	Hu et al. [140]
CCAT2	Upregulated	Unknown	Associated with poor prognosis	Ning et al. [141]

### Therapeutic strategies

Curcumin is a natural compound found in turmeric that has been shown to exhibit anti-cancer properties in various cancers, including cervical cancer. Curcumin increases the expression of MEG3 in ovarian cancer by bringing about DNA hypomethylation [142]. A combination of dendrosomal nanocurcumin and oxaliplatin was also found to alter the expression of lncRNA in ovarian cancer and promote treatment [143]. Sanguinarine had anti-tumour effect against epithelial ovarian cancer cells via the CASC2–EIF4A3 axis and/or PI3K/AKT/mTOR and NF-κB signalling pathways [144]. Resveratrol is a natural compound found in grapes, berries, and peanuts that has been shown to possess anti-cancer properties. A large microarray and in silico analysis study by Vallino et al. comprehensively showed that resveratrol could have anti-cancer effect on ovarian cancer cells by alterations of lncRNA via epigenetic modulation of various regulatory pathways including proliferation, apoptotic and migration [145]. It is shown to modulate GAS5, UCA1, LINC00092, PVT1, UCA1, HULC and MEG3 amongst several others. Ginsenoside 20 (S) -Rg3, found in red ginseng, was shown to supress the Warburg effect in ovarian cancer cells by modulating the lncRNA H19/miR-324-5p/PKM2 pathway as well as downregulating a total of 67 lncRNAs impacting various cellular processes [146]. Treatment of mice models with targeted polymers of nucleic acids against lncRNA HOTAIR, was found to reduce its expression and interaction with EZH2, leading to anti-cancer effect [147]. The treatment of ovarian cancer cells with a plasmid expressing the diphtheria toxin gene under the control of the lncRNA H19 promoter produced anti-cancer effect with adequate safety [148]. Treatment of ovarian cells with the demethylating agent, 5-azacytidine, brought about hypomethylation of lncRNA MEG3 promoter and suppresses cell proliferation [149].

MALAT1 knockdown in ovarian cancer cells resulted in upregulation of miR-218 with loss of proliferation and cell cycle arrest [150]. Knockdown of the lncRNA HOXD-AS1, reduced the migration and invasion of ovarian cells and resulted in better outcomes [151]. Cheng et al. showed that siRNA targeting of lncRNA AB073614 resulted in potent obstruction of cell proliferation, invasion and activation of apoptosis in HO-8910 and OVCAR3 ovarian cancer cell lines and in vivo studies supported these data [152]. In another study, Gordon et al. used modified ASO techniques to block the expression of MALAT-1 in anoikis-resistant ovarian cancer cell lines [132] (Table 6).

**Table 6:** Therapeutc strategies involving lncRNA in ovarian cancer

Name of the therapeutic agent	Sample	Molecular target	Effect	Reference
Curcumin	Ovarian cancer cells	MEG3 via DNA hypomethylation	Increases MEG3 expression, promotes anti-cancer effects	Zhang et al. [112]
Dendrosomal nanocurcumin + oxaliplatin	Ovarian cancer cells	Alters expression of lncRNAs	Promotes treatment efficacy	Seyed Hosseini et al. [143]
Sanguinarine	Epithelial ovarian cancer cells	CASC2–EIF4A3 axis, PI3K/AKT/mTOR, NF-κB pathways	Anti-tumour effect	Zhang et al. [110]
Resveratrol	Ovarian cancer cells	Epigenetic modulation of lncRNAs (GAS5, UCA1, LINC00092, PVT1, HULC, MEG3)	Anti-cancer effects by impacting proliferation, apoptosis, and migration	Vallino et al. [145]
Ginsenoside 20(S)-Rg3	Ovarian cancer cells	lncRNA H19/miR-324-5p/PKM2 pathway, 67 lncRNAs	Suppresses the Warburg effect, impacts various cellular processes	Zheng et al. [146]
Targeted polymers against lncRNA HOTAIR	Mice models	Reduces expression lncRNA HOTAIR, EZH2 interaction	Produces anti-cancer effects	Wu et al. [147]
5-azacytidine	Ovarian cancer cells	MEG3 promoter hypomethylation	Suppresses cell proliferation	Li et al. [149]
MALAT1 knockdown	Ovarian cancer cells	miR-218	Loss of proliferation, cell cycle arrest	Shi et al. [150]

## Role of lncRNAs and uterine cancer

### Role in carcinogenesis

Uterine cancer is a complex and multifactorial disease that arises from the abnormal growth and proliferation of cells in the uterus. It can be broadly categorized into benign tumors, such as hysteromyoma, and malignant tumors, including endometrial cancer (ES) and endometrial stromal sarcoma (ESS). ESS is in scarce type of cancer. Because of the scarceness of ESS patients (~ 0.2% of all uterine malignancies) sufficient evidence on the pathogenicity along with prognosis of this malignancy is not available. Of the various gynecological malignancy, ES is the most frequent worldwide, accounting for approximately 4% of all cancers in women. Although the exact causes of endometrial cancer are not fully understood, several risk factors have been identified, including obesity, hormonal imbalances, and genetic mutations. Based on lncRNA cluster expression, EC patients have been divided into three groups- a. Basal like comprises of aggressive tumors with the higher frequency of *PTEN* deletion, *p53* mutation, with higher manifestation of polycomb genes. Therefore, the survival rate is poorer than the other subgroups [153, 154] b. The luminal subgroup is linked with the progesterone and estrogen receptor genes expression, c. whereas the mutated *β-catenin* and *PTEN* genes are linked with β-catenin subgroup [155]. The basal-like subgroup demonstrated higher aberrant expression of different lncRNAs compared to the other two subgroups. Around 50 lncRNAs (including LINC00488) exhibited suppressed expression whereas, 33 other lncRNAs (HOTAIR), were overexpressed [153]. Therefore, based on these findings, it is possible to discriminate between aggressive and non-aggressive endometrioid EC by looking at the expression pattern of lncRNAs. Various lnRNAs exhibited differential expression in tumor suppressive lncRNAs expression were downregulated such as CASC2, GAS5, MEG3, FER1L4, and LINC00672 expression were in EC tissues in contrast to normal tissues [154, 156–159]. Tumor suppressive effect of GAS5 was evident as the higher expression of GAS5 led to in apoptosis in HHUA and JEC (EC cell lines). In HEC-1B and Ishikawa cells, stable overexpression of MEG3 dramatically caused apoptosis and decreased migration and invasion (9). Gain-of-function research on the EC cell line HEC-50 indicated that FER1L4 might reduce the growth of the cell and trigger apoptosis. The overexpression of LINC00672 in HEC-1A and Ishikawa cells decreased cell proliferation, according to a subsequent functional analysis.

In endometrial carcinoma, oncogenic lncRNA (CCAT2, BANCR, NETA1) were found to upregulated compared to the normal endometrial tissues. CCAT2 (HEC-1-A and RL95-2 cells) silencing and knockdown of BANCR (HEC-1A cells) and NEAT1 (HEC-59) suppressed cell proliferation, migration and invasion [160–162]. The steroid receptor RNA activator (SRA) gene mutation is observed to have higher expression in steroid-rich tissues, such as the brain, but is expressed at low levels in normal uterine and ovarian tissues. However, studies have shown that SRA is upregulated in ovarian and uterine tumors, suggesting a potential role in cancer development. Ectopic expression of SRA alone in male accessory sex glands and the epithelial cells nuclei of the mammary gland is inadequate to cause tumorigenesis in animal models, however, it has been demonstrated to block Ras-induced carcinogenesis [163, 164].

Another important gene in the context of uterine carcinoma is the MALAT-1 (metastasis-associated lung adenocarcinoma transcript 1) gene [153]. MALAT-1 is upregulated in some types of uterine cancers, and studies have suggested that it may act as a biomarker for distinguishing between proliferative and secretory phases of endometrial cancer as expression patterns for MALAT-1 was similar in both ESS tumor cells and normal stromal cells during proliferation phase but in the secretory phase it was substantially lower. Furthermore, high levels of MALAT-1 were reported to be linked with increased risk of ESS relapse and metastasis [165, 166]. The preferential epigenetic silencing of one of the paternal alleles is called genomic imprinting. H19, one of the first lncRNAs to be discovered [153], works in genomic imprinting during cell development by being upstream of insulin-like growth factor (IGF 2). The imprinted and developmentally regulated H19 gene has been associated to a range of human cancers, although the underlying mechanisms are still poorly understood. H19 was shown to be elevated in EC and linked to EC advancement, according to Doucrasy et al. [167]; the amount of H19 in the myometrium and stroma was linked to the cell propagation rate. In addition to genetic mutations, other factors may cause development of uterine cancer. For example, the H19 gene has been identified as a potential biomarker for endometrial stroma in endometrial atypical hyperplasias. H19 is not detected in normal endometrieal tissue but is expressed in hyperplastic and neoplastic epithelium. Its expression frequency is also relatively high in stromal cells of normal endometrium, suggesting a potential role in cancer development [167, 168].

Cell growth and motility in EC is regulated by downregulating tumor-suppressive microRNA i.e., let 7, that reduces the expression of oncogenes (shigh mobility group c-Myc, AT-hook 2, and IGF2 binding protein 3) post-transcriptionally. Yan et al. reported that H19 facilitates invasive and migratory ability in cancer cells. In in vivo condition a mechanism has revealed which is independent of the H19–let–7 axis in EC and ovarian cancer which results in the co-expression of H19 and certain oncogenes [167, 168]. H19 is not detected in normal endometrial tissue but is expressed in hyperplastic and neoplastic epithelium[167, 168]. The study also showed that metformin, an anti-diabetic medicine, might reduce cancer cells' ability to migrate, in part because of hypermethylated impacted H19 downregulation. These findings explain H19 impacted metastasis and also the justifies why elevated expression of let7 (tumor suppressor) are unexpectedly linked to a poor prognosis in certain instances [167].

Additionally, the categorization of lncRNA subtypes in EC was subjected to the first thorough characterization investigation [168] and in cancer a total of 53 lncRNAs with distinct expression pattern was found compared to healthy endometrial tissue and they were found to be linked to various different physiological processes, molecular processes and expression was small nucleolar RNA host gene 12 (ASLNC04080), which may advance EC via co-regulating with protein-coding genes. In HEC1B endometrial cancer cells, the downregulation of ASLNC04080 deterred cell proliferation as well as enhanced apoptosis and led to G1 phase arrest [169].

The amount of tumor aggressiveness in EC may be influenced by the OVAL (ovarian adenocarcinoma amplified lncRNA), [170]. Akrami et al. discovered that OVAL displayed limited focused genomic amplification in some kinds of cancer tissue by looking for areas of copy-number changes that lack protein-coding targets. Serous EC and additional carcinoma types shared similar genomic amplification characteristics; hence, OVAL might also be considered as biomarker to distinguish between type I and type II EC [168, 170]).

Another important lncRNA, HOTAIR located on chromosome 12 and regulates the expression of chromosome 2 genes and regresses the expression of HOXD genes [168]. HOTAIR is observed to be upregulated in different cancers and is demonstrated to be negatively involved in regulating metastasis-suppressing genes which induces tumor malignancy [171]. Likewise, in EC also the expression of HOTAIR is higher in comparison to the normal endometrial tissue and the level of expression is correlated with the clinical stage of the cancer [172]. Previously an in vivo study demonstrated that knocking down of HOTAIR suppresses EC tumorigenesis through inhibiting cell propagation and metastasis [155].

In several types of cancers MALAT1 has been shown to be a potential biomarker including cervical cancer, uterine endometrial stromal sarcoma. This is because MALAT1 is associated with metastasis and in non-small cell lung carcinoma, MALAT1 is being considered as a target for anti-metastatic therapy. In uterine endometrial carcinoma (EC), the regulatory axis Wnt/β-catenin-PCDH10-MALAT1 may be involved in development of the disease. However, the exact mechanism employed by this axis in diverse pathological conditions linked to EC is not fully understood. Nonetheless, MALAT1 is being investigated as a potent biomarker and target for therapeutic intervention in EC [166].

Another lncRNA called HOTAIR linked with the invasion of the muscle layer of the uterus and migration of cancer cells to the lymph nodes in EC. Higher expression of HOTAIR is linked to the overall reduced survival time was with compared with the patients with lower expression. In EC, inhibiting HOTAIR through RNA interference showed promising therapeutic strategy. It is pertinent to note that targeting a single lncRNA can act multi-directionally by interfering with the multiple pathways simultaneously. As HOTAIR is correlated with the EC progression therefore may act as suitable therapeutic target and a potential biomarker for poor prognosis [173] (Table 7).

**Table 7:** Role of lncRNA in uterine cancer

lncRNA	Expression	Target	Effect	References
HOTAIR	Upregulated	HOXD genes, Wnt/β-catenin pathway	Promotes cell proliferation, migration, invasion, linked to advanced clinical stages of cancer	(Li and Wan [168]; Huang et al. [172]; He et al. [196])
GAS5	Downregulated	Apoptosis pathways in HHUA and JEC cells	Induces apoptosis, inhibits proliferation	(Jiang et al. [154]; Qiao and Li [158])
MEG3	Downregulated	Apoptosis and migration/invasion pathways in HEC-1B and Ishikawa cells	Induces apoptosis, reduces migration and invasion	(Li et al. [156]; Sun et al. [157])
FER1L4	Downregulated	Unknown	Reduces cell growth, triggers apoptosis	(Kong and Ren [159])
LINC00672	Downregulated	Unknown	Decreases cell proliferation	(Li et al. [156]; Sun et al. [157])
CCAT2	Upregulated	Unknown	Promotes cell proliferation, migration, and invasion	(Li et al. [165])
BANCR	Upregulated	Unknown	Promotes cell proliferation, migration, and invasion	(Wang et al. [161])
NEAT1	Upregulated	Unknown	Promotes cell proliferation, migration, and invasion	(Xie et al. [160])
SRA	Upregulated	Steroid receptor RNA activation	Blocks Ras-induced carcinogenesis in animal models	(Zhao et al. [164]; Liu et al. [197])
MALAT1	Upregulated	Wnt/β-catenin-PCDH10-MALAT1 axis	Associated with metastasis and poor prognosis, potential biomarker for therapy	(Qiao et al. [166])
H19	Upregulated	let-7 tumor-suppressor microRNA, IGF2	Facilitates migration and invasion, linked to cancer cell aggressiveness	(Doucrasy et al. [167]; Li and Wan [168])
ASLNC04080	Downregulated	Protein-coding genes	Inhibits cell proliferation, induces apoptosis	(Zhai et al. [169])

Owing to the lack of of noticeable symptoms the early diagnosis of EC can be challenging. Additionally, in aggressive types of EC, such as serous adenocarcinoma, reliable biomarkers that may help in predicting prognosis is not known. Transcriptomics and proteomics approaches could not stipulate precise biomarkers. However, profiling the expression of lncRNAs rather than profiling hundreds or thousands of messengers RNAs (mRNAs) or proteins may offer a promising alternative, as the expression patterns of these molecules can provide more information about the prognosis and diagnosis markers. However, further research is needed to explore the involvement of lncRNAs in drug resistance.

### Therapeutic strategies

A study by Pandey et al. [174] have proved that Deoxyelephantopin, a novel phytochemical exhibited a potential anti-carcinogenic property against uterine leiomyoma (UL) cells and downregulated lncRNA such as HOTAIR, BANCR, H19, and ROR [174].

Considering the significance of lncRNAs in tumorigenesis different strategies have been developed, such as inhibiting the interaction of the lncRNA with its binding partners or using siRNA to knockdown the pathogenic lncRNA, [153, 175, 176]. Various studies both in vitro and in vivo have advocated the use of RNA interference strategy target oncogenic lncRNAs such as H19, HOTAIR, BANCR, and ASLNC04080 against EC. Whereas, ectopic expression of tumor suppressor lncRNA (MEG3) demonstrated anti-cancer effect (Table 8) [169, 175, 177].

**Table 8:** Therapeutic strategies involving lncRNA in uterine cancer

Therapeutic agent	Sample	Molecular target	Effect	Reference
Deoxyelephantopin (phytochemical)	Uterine leiomyoma (UL) cells	Downregulates lncRNAs HOTAIR, BANCR, H19, and ROR	Exhibits anti-carcinogenic properties	(Pandey et al. [174])
siRNA strategy	In vitro and in vivo models	Targets oncogenic lncRNAs like H19, HOTAIR, BANCR, and ASLNC04080	Inhibits proliferation, migration,	(Parasramka et al. [175]; Qi et al. 2016; Dong et al. [153])
Ectopic expression (MEG3)	In vitro and in vivo models	Tumor-suppressor lncRNA (MEG3)	Demonstrates anti-cancer effects	(Zhai et al. [169]; Parasramka et al. [175]; Yang et al. [134])
CRISPR/Cas9 (SpCas9-HF1 variant)		High-fidelity Cas9 variant for reduced off-target effects	Exhibits anti-carcinogenic properties	(Yang et al. [177] Dong et al. [153])

The genome-editing technology CRISPR/Cas9, holds great promise as a transformative approach for developing effective anti-cancer therapies [178, 179].

Despite these advancements, genome-wide analyses have revealed that only a subset of lncRNA loci, approximately 38% out of 15,929, can be safely targeted using the CRISPR/Cas9 system. The rest of lncRNA loci may disrupt the neighboring genes, presenting a challenge in achieving selective targeting of specific lncRNA regions [179, 180]. To address this hurdle, researchers have employed innovative technique by using a high-fidelity variant of the Cas9 protein i.e., SpCas9-HF1, with an altered amino acid sequence to reduced off-target effects, hence, enhancing the precision of CRISPR/Cas9 genome editing (Yang et al. [134]; Dong et al. [151])Furthermore, the utilization of tumor-specific promoters to drive Cas9 expression offers a means to increase the specificity of CRISPR/Cas9 for cancer cells, minimizing potential impacts on healthy cells.

### Circulatory lncRNAs as biomarkers in gynecological cancers

Circulating lncRNAs in circulation have drawn interest as non-intrusive biomarkers for gynecological cancer detection and surveillance. Their therapeutic potential is highlighted due to their association in carcinogenic pathways, stability in bodily fluids, and cancer specific manifestation.

The potential of circulating long non-coding RNAs (lncRNAs) as novel biomarkers for gynecological cancers, such as breast, endometrial and ovarian tumours, has been brought to light by recent investigations. These lncRNAs, which include MALAT1, H19, HOTAIR and others, have been found to be important contributors to the development and spread of cancer. They are reported to be useful for early identification and therapy response surveillance. Few important Circulatory lncRNAs as biomarkers and their roles are as follows.

- H19: Elevated in ovarian and endometrial cancers, H19 correlates with tumor proliferation, metastasis, and chemoresistance. Its levels drop post-tumor resection, underlining its diagnostic utility (Zhang et al., [195])
- MALAT1: Known to promote epithelial-mesenchymal transition (EMT) in ovarian cancer, circulating MALAT1 serves as a marker of aggressive disease and poor prognosis. Its role in modulating angiogenesis also makes it a therapeutic target (Wang et al., [48]).
- HOTAIR: Found in high levels in endometrial and cervical, breast cancers. circulating HOTAIR facilitates chromatin remodelling and metastasis. Its presence in serum has been linked to advanced disease stages and reduced survival rates (Liu et al., [109, 113]).
- PCA3: Although initially identified in prostate cancer, PCA3 has emerged as a candidate biomarker for endometrial cancer, with its urinary levels reflecting disease progression.
- CCAT2: Upregulated in cervical cancer, CCAT2 regulates the Wnt/β-catenin pathway, promoting invasion and metastasis. Circulating CCAT2 levels show promise in distinguishing cancer patients from healthy individuals (Cao X [194]).

Although early detection and surveillance of lncRNAs in liquid biopsies can transform the face of the personalized cancer treatment, however, further studies related to their molecular mechanistic are warranted to augment their use in clinical settings.

## Limitations and challenges of lncRNA-based therapeutics

Despite the fact that lncRNA-based treatments is a promising therapeutic strategy for a number of illnesses, including cancer, there are a number of limitations that need to be resolved, which are as follows-

The difficulty associated with the distribution of lncRNA-targeting medicines effectively to the required tissues or cells. Compared to tiny molecules or conventional medications, the distribution of lncRNAs is more difficult due to their enormous size and structural complexity. For a treatment to be successful, it is imperative to create efficient delivery methods that can prevent lncRNAs from being degraded and promote their absorption into target cells [182]. As lncRNAs play complex regulatory roles in the genome, altering them may unintentionally influence other biological processes. Off-target effects, where one lncRNA's modification may affect the expression or operation of other genes or pathways, are a possibility. The safe and successful use of lncRNA-based therapies depends on ensuring specificity and reducing off-target effects [183, 184]. The specific molecular processes and functional functions of many lncRNAs are still poorly known, despite advances in lncRNA research. The creation of tailored treatments is hampered by the lack of understanding of lncRNA interactions and functions. To maximize the therapeutic potential of certain lncRNAs, more study is required to clarify their roles and modes of action [185]. Pharmacokinetics and Stability: LncRNAs can have brief half-lives in the body and are subject to nuclease-mediated destruction. To achieve adequate exposure and long-lasting effects in the target tissues, stability and pharmacokinetic aspects of lncRNA-based therapies need to be carefully considered [184]. The regulatory environment for therapies based on lncRNA is currently developing. To ensure the safety, effectiveness, and manufacture of lncRNA therapies, there must be defined regulatory norms and recommendations. Clinical translation of lncRNA-based therapeutics depends on addressing regulatory issues and securing required clearances.

Preclinical investigations have yielded encouraging findings, but thorough validation through carefully planned clinical trials is still required before lncRNA-based treatments can be translated into clinical practice. Large-scale clinical trials must be carried out, suitable patient populations must be established, biomarkers must be established, and clinical efficacy must be proven for lncRNA-based medicines to be developed and adopted [183, 186, 187]. Therefore, for success of development and application of lncRNA-based therapies as efficient treatment alternatives for diverse illnesses, it is important to address these constraints and challenges.

## Isoforms of LncRNA

LncRNA genes can undergo alternative splicing, producing different lncRNA isoforms. However, the biological roles of most lncRNA isoforms remain largely unexplored, with research primarily concentrating on variants that exhibit relatively high expression levels.

The NEAT1 gene encodes two transcriptional isoforms: a shorter variant, NEAT1-1, and a longer variant, NEAT1-2. NEAT1 serves as a scaffold for various RNA-binding proteins (RBPs), paraspeckle formation, guidance, and competing endogenous RNA (ceRNA) activity. As a ceRNA, NEAT1 sequesters miRNAs, preventing them from interacting with their downstream targets. Additionally, as a guide, NEAT1 interacts with RNA-binding proteins to facilitate their precise localization to specific gene loci [55].

A 5.7 kb isoform of Malat1 (Malat1_2), derived from a conserved region and possibly regulated by a hidden internal promoter within the Malat1 locus, acts as a crucial antioxidant in in vivo animal models. It protects the brain and retina from age-related cellular senescence, highlighting a distinct physiological role for the lncRNA Malat1, unrelated to its oncogenic functions [188].Another study by shower that MALAT1 was overexpressed in 14% of breast tumors, with epithelial cells showing nuclear signals and large speckles. The alternatively spliced transcript Δsv-MALAT1, underexpressed in 18.8% of cases, was identified as an independent prognostic factor. Its expression was linked to alterations in RNA splicing machinery, the Drosha-DGCR8 complex, YAP protein status, and activation of the PI3K-AKT pathway [189].

Recent studies indicate that Cold atmospheric plasma treatment induces opposite expression patterns of ZNRD1 and its antisense lncRNA ZNRD1-AS1 in breast cancer cells, depending on treatment conditions [190].

## Future direction of lncRNA-based therapeutics

Several potential future paths hold promise for enhancing the development and use of lncRNA-based treatments since the area of lncRNA-based therapeutics is continuously evolving. Enhanced Delivery methods: A major area of study is on enhancing delivery methods for lncRNA-based therapies. The effectiveness and specificity of delivering lncRNAs to target tissues or cells can be enhanced by developments in nanoparticle-based delivery methods, viral vectors, exosome-mediated delivery, and tailored delivery strategies. For clinical translation to be effective, delivery devices that can efficiently get over obstacles and distribute lncRNAs to target areas will be essential [184].

Investigating combinations of chemotherapy, immunotherapy, or targeted treatments with lncRNA regulation has potential for synergistic effects and enhanced therapeutic results. Combining lncRNA-based treatments with currently used therapeutic modalities may increase their effectiveness, get around resistance mechanisms, and offer individualized treatment alternatives [35]. Patient stratification and individualized treatment strategies can be aided by the identification and validation of particular lncRNA biomarkers linked to illness diagnosis, prognosis, or therapy response. The creation of trustworthy, non-invasive methods to identify and gauge the levels of lncRNA expression in patient samples may help in the early diagnosis of illness, the monitoring of treatment outcomes, and the direction of therapy choices [187]. Genome editing tools provide promise for precise and effective therapeutic treatments when used to target the modification of lncRNAs, such as CRISPR-Cas9. LncRNA-based treatments may have novel applications if methods for editing lncRNA sequences or altering their activities through epigenetic changes are developed. Using CRISPR/Cas9 technology opens up a viable new path for targeted cancer treatment. However, difficulties with the secure targeting of lncRNA regions continue to exist. The key to realizing the full potential of CRISPR-based medicines in the battle against cancer therefore lies in continued study and improvement of these approaches [35, 187].

The identification, annotation, and prediction of lncRNA activities and interactions can be aided by developments in bioinformatics and computational techniques. Deciphering the intricate functions of lncRNAs in illnesses and facilitating the design of tailored therapeutic treatments can be made easier by integrating multi-omics data, network analysis, and machine learning methodologies. Using CRISPR Effectors to Target RNA (RNA-Targeting CRISPR Systems): LncRNAs may be selectively targeted and destroyed using RNA-targeting CRISPR systems like Cas13 and CasRx. These technologies provide a cutting-edge method for lncRNA regulation by using the target-recognition capabilities of CRISPR effectors. In addition to altering endogenous lncRNAs, scientists are working on creating synthetic lncRNA mimics that can act in a manner like a given lncRNA. A novel strategy for lncRNA-based therapies is made possible by the capacity to build these synthetic constructs to have increased stability, specificity, and delivery qualities [191, 192]. Validating the therapeutic effectiveness and safety of lncRNA-based medicines will depend on conducting well-designed clinical studies with bigger patient populations, specified outcomes, and suitable control groups. The development of lncRNA-based medicines into clinical practice depends on partnerships between regulatory agencies, industry, and academia. These future prospects provide great opportunities for the creation of efficient and precise therapies for a variety of disorders as research in the field of lncRNA-based therapeutics advances.

## Conclusion

In conclusion, although there have been significant advances in the study of lncRNAs that open fresh possibilities for the clinical diagnosis and better management of cancer. However, there are still many difficult obstacles to deal with and overcome such as Further research is needed to fully understand the roles and regulation of lncRNAs in health and disease. Due to the lack of effective and convenient detection methods for detecting lncRNAs in the circulatory system, its clinical application as the potential diagnostic markers is hindered. lncRNAs have the potential to serve as important biomarkers and diagnostic tools in cancer. Further research is needed to validate these findings and develop standardized assays for the clinical use of lncRNAs in cancer diagnosis and management.

## Data Availability

No datasets were generated or analysed during the current study.
